# Poly(Methyl Methacrylate) Coatings Containing Flame Retardant Additives from Suspensions in Water-2-Propanol

**DOI:** 10.3390/molecules26071974

**Published:** 2021-03-31

**Authors:** Xuelin Li, Zhengzheng Wang, Sadman Sakib, Ritch Mathews, Igor Zhitomirsky

**Affiliations:** 1 Department of Materials Science and Engineering, McMaster University, Hamilton, ON L8S4L7, Canada; lix387@mcmaster.ca (X.L.); wangz338mcmaster.ca (Z.W.); 2 Department of Engineering Physics, McMaster University, Hamilton, ON L8S4L7, Canada; sakibs@mcmaster.ca; 3 Advanced Ceramics Corporation, 2536 Bristol Circle, Oakville, ON L6H5S1, Canada; rmathews@acc.ca

**Keywords:** poly(methyl methacrylate), huntite, halloysite, hydrotalcite, hydroxide, coating

## Abstract

A dip-coating technique is designed for deposition of poly(methyl methacrylate) (PMMA) from water/2-propanol mixture, avoiding the use of traditional toxic solvents. Solutions of PMMA macromolecules with high molecular weight (M_W_) are obtained for a water/2-propanol ratio of 0.15–0.33 and the solubilization mechanism is discussed. The ability to use concentrated PMMA solutions and high M_W_ of the polymer are the key factors for the successful dip coating deposition. The coating mass for 10 g L^−1^ polymer solutions shows a maximum at a water/2-propanol ratio of 0.25. The deposition yield increases with the polymer concentration increase and with an increasing number of the deposited layers. PMMA deposits protect stainless steel from aqueous corrosion. The coating technique allows for the fabrication of composite coatings, containing flame-retardant materials (FRMs), such as commercial halloysite, huntite, hydrotalcite, and synthesized Al(OH)_3_, in the PMMA matrix. The FRM content in the coatings is modified by variation of the FRM content in colloidal suspensions. A fundamentally new method is developed, which is based on the salting out aided dispersive extraction of Al(OH)_3_ from the aqueous synthesis medium to 2-propanol. It is based on the use of hexadecylphosphonic acid molecules as extractors. The method offers advantages of reduced agglomeration.

## 1. Introduction

Poly(methyl methacrylate) (PMMA) coatings are widely used for protection of metals from aqueous corrosion, surface modification of biomedical implants, fabrication of solar cells, batteries and supercapacitors, optical devices, and biosensors [1]. PMMA exhibits good chemical stability and mechanical properties, biocompatibility, and thermal stability [1]. The selection of solvents for PMMA is important for coating deposition techniques, such as electrophoretic deposition, dip coating, spin coating, and spray-coating. PMMA is well-soluble in benzene, toluene, and methyl ethyl ketone, which are carcinogenic and toxic. Therefore, there is a need to avoid the use of such solvents for the fabrication of PMMA coatings.

Flame retardant materials (FRMs) are usually added to PMMA and other polymers to meet safety regulations [2]. However, many common FRMs, such as halogen- and phosphate-based FRMs, are toxic and bioaccumulative [2,3]. Investigations showed that the use of halogen-based FRMs resulted in widespread contamination of the environment [4,5]. Therefore, halogenated FRMs must be replaced by environmentally friendly FRMs [2].

Natural minerals, such as halloysite, huntite, and hydrotalcite, are promising FRMs. Aluminum hydroxide is a low-cost FRM additive for various FR applications. The FR properties of such materials are attributed to endothermic decomposition and release of H_2_O or CO_2_, which dilute fuel. Thermal analysis of halloysite showed mass loss of about 18% related to dehydration and related endothermic effect [6,7]. Halloysite is an important FRM additive for different polymers [6,7]. Natural halloysite forms nanotubes, which can reinforce polymer materials [7,8]. Moreover, halloysite nanotubes can be loaded with other functional materials, such as corrosion inhibitors [9]. Huntite is another advanced FRM additive for different polymers, because it is a naturally available mineral [10,11,12,13]. It is a halogen-free, non-corrosive, and recyclable FRM [10]. Many investigations focused on the analysis of thermal behavior of huntite [10,12,13], which showed mass loss related to CO_2_ gas release of about 50%. The relatively high mass loss related to thermal decomposition of huntite and a significant endothermic effect are important factors for the FRM applications of this material. Huntite has a relatively high thermal decomposition temperature [12,13], which is higher than the processing temperatures of important thermosetting polymer products. Therefore, huntite can be added to thermosetting polymers. The interest in the fabrication of polymer composites containing hydrotalcite is attributed to the excellent FRM properties of this material, such as significant water release due to its thermal dehydration and the related endothermic effect. Many investigations focused on the analysis of thermal properties of hydrotalcite at different conditions and fabrication of polymer-hydrotalcite composites [14,15,16]. It was found that thermal decomposition of hydrotalcite results in mass loss of 43%, which is related to the release of H_2_O and CO_2_. Aluminum hydroxide decomposition results in mass loss of 35%, which is solely related to H_2_O release. In contrast to huntite and hydrotalcite, the release of CO_2_ can be avoided. The significant water release related to its thermal dehydration makes Al(OH)_3_ a promising material for many FRM applications [17,18]. Previous investigations highlighted the need for a uniform distribution of non-agglomerated Al(OH)_3_ nanoparticles in a polymer matrix for the deposition of high-quality composites, containing Al(OH)_3_ as an FRM additive.

The objective of this work was the deposition of PMMA and composites, containing inorganic FRM additives, such as halloysite, huntite, hydrotalcite, and Al(OH)_3_, avoiding the use of carcinogenic and toxic organics, such as organic solvents and organic FRM additives. FRMs are mandatory additives for various polymer products for the reduction of polymer flammability. Inorganic FRM additives represent a promising alternative to toxic halogenated FRMs. Moving towards the objective of this work, we analyzed the PMMA solubility in water-2-propanol co-solvent with different water contents and optimized the deposition yield in various experimental conditions. We developed a fundamentally new approach, which involved synthesis of Al(OH)_3_ nanoparticles by an aqueous precipitation method, their salting out aided dispersive extraction to 2-propanol, and fabrication of Al(OH)_3_ dispersions in PMMA solutions using water-2-propanol for coating application. The method facilitates agglomerate-free processing of hydroxide nanoparticles. It is conceptually different from other particle extraction techniques [19] and can potentially be used for dispersive extraction of various functional materials.

## 2. Results and Discussion

PMMA is insoluble in water and 2-propanol at room temperature. However, we found that high-molecular-mass PMMA was soluble in different water-2-propanol mixtures with a water/2-propanol ratio of R_wip_ = 0.15–0.33. First, 10 g L^−1^ PMMA solutions were prepared using solvent compositions in the range of 0.15 < R_wip_ < 0.33. However, PMMA precipitation was observed at R_wip_ < 0.15 and R_wip_ > 0.33. The highest PMMA solubility of 20 g L^−1^ was achieved at R_wip_ = 0.25. However, the solubility decreased with decreasing or increasing R_wip_.

The ability to form relatively concentrated PMMA solutions using a high M_W_ polymer was crucial to the PMMA deposition by dip-coating and avoiding the use of carcinogenic and toxic organic solvents. Water-alcohol mixtures were used as co-solvents for low M_W_ PMMA [20] at essentially lower polymer concentrations. However, low M_W_ PMMA exhibits poor adhesion and inferior ability to form good-quality coatings. Usually, the increase of polymer M_W_ leads to decreasing solubility due to stronger interactions of larger polymer macromolecules.

**Figure 1:**
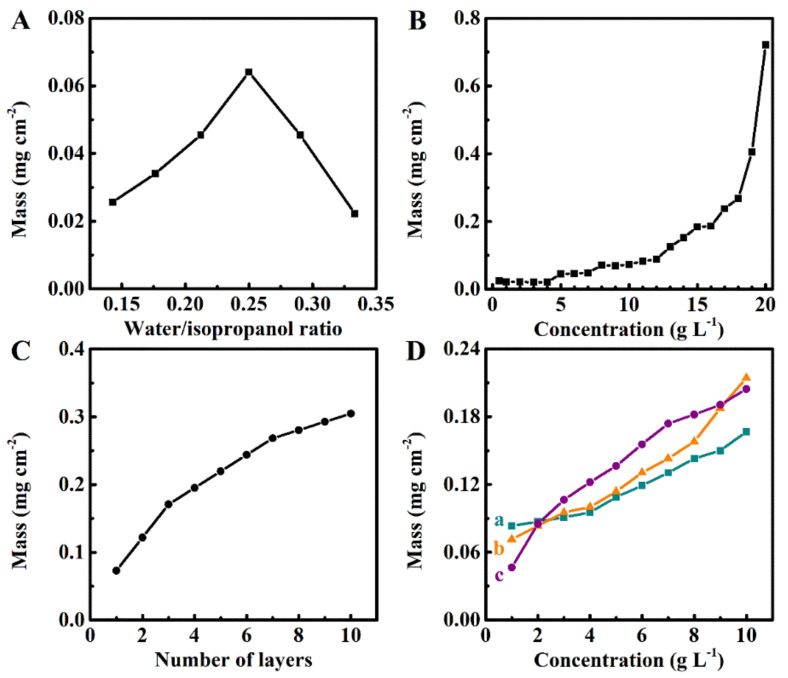
(**A**) Coating mass versus water/2-propanol ratio (R_wip_) at a poly(methyl methacrylate) (PMMA) concentration of 10 g L^−1^, (**B**) coating mass versus PMMA concentration in a solution with R_wip_ = 0.25, (**C**) coating mass versus number of individual layers deposited from 10 g L^−1^ PMMA solutions with R_wip_ = 0.25, (**D**) coating mass versus the concentration of commercial flame-retardant materials (FRMs): (**a**) halloysite, (**b**) huntite, and (**c**) hydrotalcite in 10 g L^−1^ solutions of PMMA for R_wip_ = 0.25.

It is known [20] that water and alcohols contribute to PMMA solvation because they interact with different structural groups of the polymer. At a low H_2_O content, individual H_2_O molecules can be involved in the H-bonding to the PMMA carbonyl ligands [20]. The increase in H_2_O content leads to H_2_O clustering, where H-bonding is preferably created between individual H_2_O molecules and such clustering prevents the H_2_O bonding to the carbonyl ligands [21]. As a result, the PMMA solubility decreased for H_2_O content R_wip_ > 0.25. The PMMA precipitation at R_wip_ < 0.25 is related to poor PMMA solvation due to lower H_2_O content in the solvent [22]. The analysis of the coating mass for 10 g L^−1^ solutions of PMMA revealed a maximum at R_wip_ = 0.25 (Figure 1A). The coating mass increased with the growing concentration of PMMA in the interval of 0.5–20 g L^−1^ for R_wip_ = 0.25 (Figure 1B). The deposition yield was low at PMMA concentrations 0.5–5 g L^−1^. The highest PMMA solubility of 20 g L^−1^ was achieved only at R_wip_ = 0.25. Therefore, further investigations were performed at a PMMA concentration of 10 g L^−1^. The coating adhesion measured according to ASTM D3359-17 standard corresponded to 5B classification. PMMA films can be obtained as monolayers or multilayers. The coating mass increased with an increasing number of individual layers (Figure 1C). Therefore, the mass of the deposits can be changed by variation of R_wip_, concentration of PMMA, and number of individual layers. FRM materials were added to the PMMA solutions and obtained colloidal suspensions were applied for the deposition of composites. The FRM suspensions containing FRM were stable for more than 3 days. The increase of coating mass with increasing FRM concentration (Figure 1D) indicated the formation of composite coatings with different FRM contents. A significant increase in the coating mass with an increasing FRM concentration at a constant PMMA concentration in the slurry indicated that coatings with an FRM content in a wide range can be prepared.

**Figure 2:**
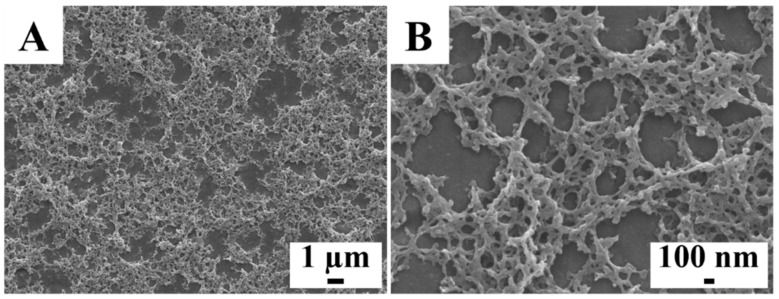
(**A**,**B**) SEM images at different magnifications of a coating fabricated using 10 g L^−1^ solution of PMMA with R_wip_ = 0.25.

Figure 2 presents SEM images of a monolayer PMMA coating prepared from 10 g L^−1^ solutions of PMMA at R_wip_ = 0.25. The deposition technique facilitated the fabrication of crack-free layers, which contained a relatively dense bottom layer and a porous top layer. The coating thickness was ~2 μm. The PMMA deposits showed protection of stainless steel from aqueous corrosion.

**Figure 3:**
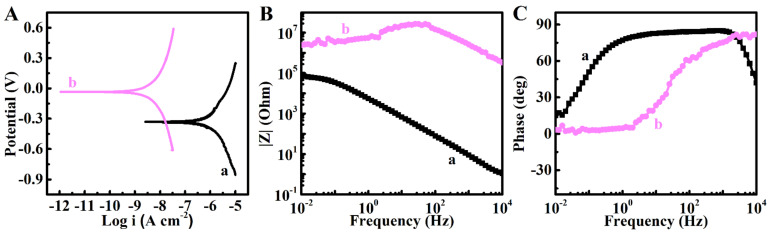
(**A**) Tafel dependences, and (**B**,**C**) Bode graphs for (**a**) uncoated substrate and (**b**) substrate coated with PMMA. The coating was prepared from 10 g L^−1^ solution of PMMA with R_wip_ = 0.25. Testing was performed in aqueous 3% NaCl using saturated calomel electrode as a reference.

Figure 3 shows the results of potentiodynamic and electrochemical impedance spectroscopy (EIS) investigations. The substrates containing the deposited PMMA layer showed a lower corrosion current (*i*_c_) and higher corrosion potential. It was found that *i*_c_ was 2.6 μA cm^−2^ for the uncoated substrate and *i*_c_ reduced to 0.025 μA cm^−2^ after PMMA deposition. EIS testing (Figure 3) revealed that the PMMA layer served as a barrier, limiting ion access to the surface. Coated samples exhibited essentially higher impedance |Z| (Figure 3B) than uncoated substrates due to high electrical resistance and low electrical capacitance of the PMMA layer. In contrast, the uncoated substrates showed low |Z| due to low resistance and high capacitance of the electrical double layer.

**Figure 4:**
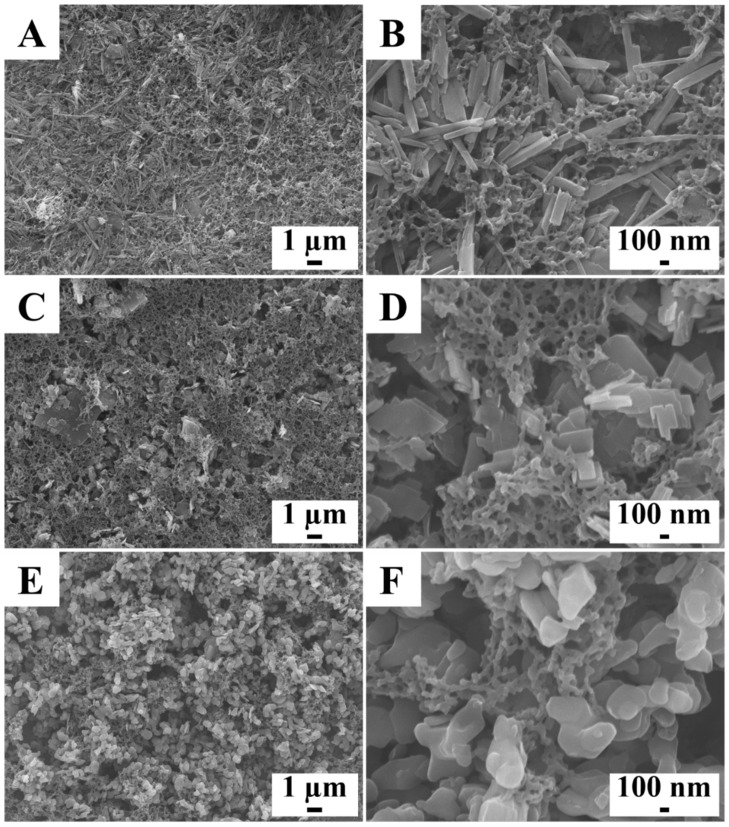
SEM images for (**A**,**B**) PMMA-halloysite, (**C**,**D**) PMMA-huntite, and (**E**,**F**) PMMA-hydrotalcite coatings, prepared from 10 g L^−1^ solutions of PMMA with R_wip_ = 0.25, containing 10 g L^−1^ FRM.

**Figure 5:**
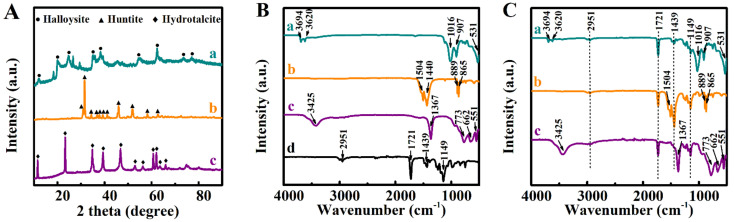
(**A**) X-ray diffraction patterns of the deposits obtained from 10 g L^−1^ solutions of PMMA with R_wip_ = 0.25, containing 10 g L^−1^ FRM additives: (**a**) halloysite, (**b**) huntite, and (**c**) hydrotalcite, (**B**,**C**) FTIR spectra of (**B**) as-received materials: (**a**) halloysite, (**b**) huntite (**c**) hydrotalcite, and (**d**) PMMA; (**C**) coating prepared from 10 g L^−1^ solutions of PMMA with R_wip_ = 0.25, containing 10 g L^−1^ FRM additives: (**a**) halloysite, (**b**) huntite, (**c**) hydrotalcite.

SEM studies (Figure 4) showed that composite coatings were deposited from the 10 g L^−1^ solutions of PMMA with 1–10 g L^−1^ FRM. The increase in FRM content above 5 g L^−1^ led to significant porosity of the composite coatings. Figure 4 presents SEM data for the deposits formed from the 10 g L^−1^ solutions of PMMA with 10 g L^−1^ FRM. The SEM images at low magnification show the deposition of crack-free layers. The images at high magnification show halloysite nanotubes (Figure 4B), huntite platelets (Figure 4D), and submicrometre hydrotalcite particles (Figure 4F). The porosity at high FRM concentrations was a result of the packing of the FRM particles. Similar to other colloidal techniques [23,24], the rugosity of the coatings was influenced by the size of particles used. The incorporation of the FRM into the PMMA coating was also supported by XRD and FTIR studies. XRD studies of the composite coating (Figure 5A) showed peaks corresponding to JCPDS files 09-0453, 14-0409, and 50-1684 of halloysite, huntite, and hydrotalcite, respectively (Figure 5A). The FTIR spectrum of PMMA (Figure 5B) and spectra of the composite materials (Figure 5C) showed peaks at 1149 cm^−1^ due to symmetric C-O-C stretching, 1439 cm^−1^ due to CH_2_ bending, 1721 cm^−1^ due to C = O stretching, and 2951 cm^−1^ due to asymmetric CH_3_ stretching [25]. The FTIR data for as-received halloysite (Figure 5B) and PMMA-halloysite (Figure 5C) showed broad absorptions at 1016, 907, and 531 cm^−1^ assigned to stretching mode Si-O-Si, bending vibrations of Al-OH, and bending and stretching vibrations of Si-O-Al [26], respectively.

The spectra of huntite (Figure 5B) and PMMA-huntite (Figure 5C) showed characteristic absorptions [13] at 865 and 889 cm^−1^, attributed to carbonate ligands. The spectra of hydrotalcite (Figure 5B) and PMMA-hydrotalcite (Figure 5C) showed peaks at 1367 cm^−1^ related to asymmetric stretching of the carbonate and at 551, 662, and 773 cm^−1^ related to oxygen–metal–oxygen stretching [27].

The ability to use water-2-propanol mixtures for the fabrication of PMMA solutions was one of the key factors for the development of a new approach for the fabrication of composite coatings. Another factor was the ability to separate 2-propanol from its mixture with water by a salting out method [28,29]. In this work, Al(OH)_3_ was prepared by a chemical precipitation from an aqueous solution of Al_2_(SO_4_)_3_. The obtained powder was washed, dried, and studied by XRD. It was found that the obtained powder was amorphous and contained agglomerated particles. In a previous investigation [19], different agglomeration mechanisms related to the drying stage were described and strategies were developed for the avoiding drying stage and transferring nanoparticles formed in water to a water immiscible organic phase via the liquid–liquid boundary. The major driving forces for agglomeration during the drying stage are reduction of the surface area of the particles and condensation of the surface OH groups, which results in oxygen bridges between the particles. Therefore, drying of the Al(OH)_3_ particles prepared in an aqueous media resulted in significant particle agglomeration. It is challenging to redisperse such particles in the coating processing media. Previous investigations [19] demonstrated the possibility of reduced agglomeration of particles by their surface modification and direct transfer from aqueous suspensions to an organic suspensions for coating or device fabrication.

**Figure 6:**
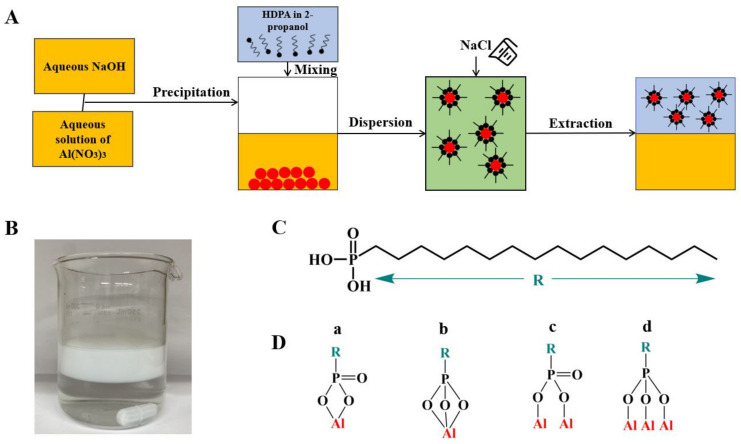
(**A**) Synthesis and salting out dispersive extraction of Al(OH)_3_ particles, (**B**) extraction of HDPA dispersed Al(OH)_3_ particles to 2-propanol, (**C**) HDPA structure, (**D**) HDPA adsorption mechanisms, involving (**a**,**b**) chelating or (**c**,**d**) bridging.

The approach developed in this investigation is conceptually different from other particle extraction techniques [19]. It involves the application of 2-propanol as an organic solvent, which was mixed with water (Figure 6). The addition of 2-propanol, containing dissolved hexadecylphosphonic acid (HDPA), to the aqueous suspension of as-precipitated Al(OH)_3_ particles allowed particle modification and dispersion in a mixed water-2-propanol solvent. The modification of particles in bulk suspensions is more efficient, compared to the modification at the interface of immiscible liquids [19], where particles must be transferred to the liquid–liquid interface from an aqueous phase, whereas extractors must be transferred to the interface from an organic phase. In our investigation, HDPA was used as a dispersant and extractor for the Al(OH)_3_ particles. The addition of NaCl allowed the separation of the 2-propanol phase, containing dispersed Al(OH)_3_ nanoparticles, from their suspension in the water-2-propanol mixture (Figure 6A,B). It is important to note that in experiments performed without HDPA, the Al(OH)_3_ particles remained in the aqueous phase. Therefore, the surface functionalization of Al(OH)_3_ particles with HDPA was critically important for their transfer to 2-propanol. Moreover, the adsorbed HDPA facilitated the fabrication of stable Al(OH)_3_ suspension in 2-propanol (Figure 6B). The chemical structure of HDPA is presented in Figure 6C. It contains phosphonate and hydrocarbon groups. It is known [30] that the phosphonate group facilitates strong bonding to the metal surface atoms of the inorganic particles by chelating or bridging bonding mechanisms (Figure 6D). The hydrocarbon group of adsorbed HDPA facilitated particle dispersion and transfer to 2-propanol.

**Figure 7:**
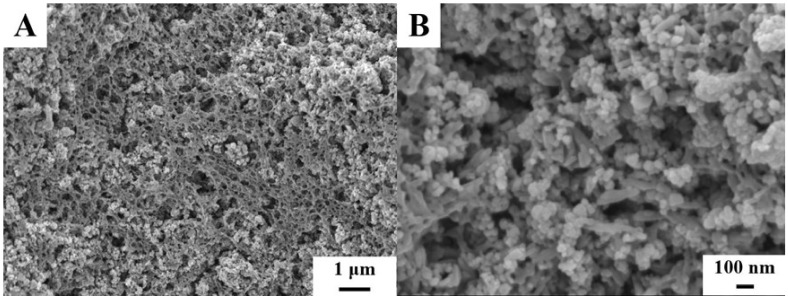
(**A**,**B**) SEM images at different magnifications for the PMMA-Al(OH)_3_ coatings, prepared from 10 g L^−1^ solutions of PMMA with R_wip_ = 0.25, containing 5 g L^−1^ Al(OH)_3_ using salting out aided dispersive extraction of Al(OH)_3_ particles.

The obtained Al(OH)_3_ dispersion in 2-propanol was used for the fabrication of Al(OH)_3_ dispersion in a mixed water-2-propanol solvent (R_wip_ = 0.25) containing dissolved PMMA. Such suspensions were utilized for the fabrication of PMMA-Al(OH)_3_ coatings. The formation of composite coatings was confirmed by SEM and FTIR. SEM studies (Figure 7A) showed that the surface layer of the coatings exhibited a porous fibrous microstructure, similar to that observed for pure PMMA (Figure 2A). However, at higher magnification (Figure 7B), nanoparticles of Al(OH)_3_ were observed with a typical size of 30–50 nm. The particles showed very low agglomeration due to the salting out dispersive extraction method developed in this investigation.

**Figure 8:**
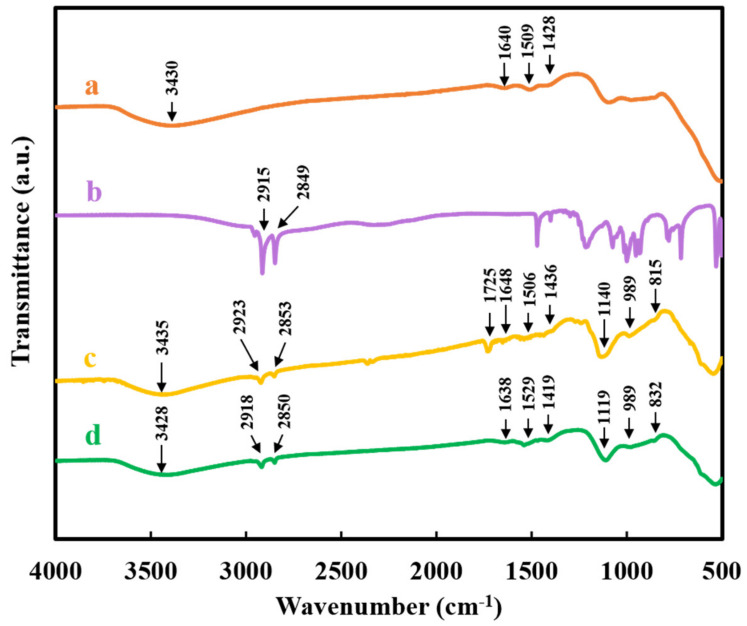
FTIR spectra of the (**a**) as-precipitated Al(OH)_3_, (**b**) as-received HDPA, (**c**) PMMA-Al(OH)_3_ coating, and (**d**) Al(OH)_3_ extracted using HDPA.

Figure 8 presents the FTIR spectra of the as-precipitated Al(OH)_3_, as-received HDPA, composite PMMA-Al(OH)_3_ coating, and extracted Al(OH)_3_. The FTIR spectrum of the as-precipitated Al(OH)_3_ shows a broad absorption at 3430 cm^−1^ due to hydroxyl stretch; small absorptions at 1640, 1509, and 1428 cm^−1^ due to bending of adsorbed H_2_O, Al-O stretching, and Al-O bending, respectively; and a broad absorption below 650 cm^−1^ due to various O-Al-O, Al-O-Al, and Al–O bending and stretching vibrations [31,32,33,34]. Similar absorptions were observed in the spectra of PMMA-Al(OH)_3_ coating and extracted Al(OH)_3_. The FTIR spectrum of HDPA showed absorptions at 2915 and 2849 cm^−1^, which were attributed to the asymmetric and symmetric stretching vibrations of CH_2_ and CH_3_ groups [35,36]. Similar absorptions in the spectra of extracted Al(OH)_3_ and composite coating confirmed the suggested extraction mechanism, which involved HDPA adsorption on Al(OH)_3_. The absorptions in the spectrum of PMMA-Al(OH)_3_ coating showed peaks at 1725 and 1140 cm^−1^, which were attributed to C = O stretching and CH_2_ bending, respectively, of the PMMA macromolecules in agreement with the FTIR data for pure PMMA presented in Figure 5.

The results of the SEM and FTIR studies confirmed the co-deposition of Al(OH)_3_ particles and PMMA. The incorporation of inorganic particles into polymer coatings is usually based on different interactions of the particles and polymers [37,38]. Investigations [39,40,41] revealed interactions of the ester group of PMMA with OH groups on the surface of inorganic particles. Therefore, such interactions can facilitate the stabilization of the Al(OH)_3_ particles in PMMA solutions in the mixed water-2-propanol co-solvent and their incorporation in the PMMA matrix to form composite coatings.

## 3. Materials and Methods

Polymethyl methacrylate polymer (PMMA, M_w_ ~ 350 kDa), Al_2_(SO_4_)_3_.18H_2_O, hexadecylphosphonic acid (HDPA), halloysite (Al_2_Si_2_O_5_(OH)_4_.2H_2_O nanotubes, length 1–2 μm, diameter 100–150 nm), hydrotalcite (Mg_6_Al_2_(CO_3_)(OH)_16_.4H_2_O, size ~ 0.5 μm), 2-propanol, and NaOH (Aldrich) and huntite (Mg_3_Ca(CO_3_)_4_ platelets, Sibelco, size ~ 0.5–3 μm) were used as starting materials. Solutions of 0.5–20 g L^−1^ PMMA in water-2-propanol with different water/2-propanol ratios were prepared at 50 °C and cooled to 20 °C. FRM materials were added to the solutions, which were then ultrasonicated for 0.5 h. The suspensions contained 10 g L^−1^ polymer and 1–10 g L^−1^ halloysite, huntite, and hydrotalcite.

The synthesis and extraction of Al(OH)_3_ was performed by dissolving 2 g of Al_2_(SO_4_)_3_.18H_2_O in 50 mL of DI H_2_O, adjusting pH to pH = 9 using NaOH, adding 100 mg HDPA in 25 mL isopropanol, stirring, and adding 6 g NaCl. The Al(OH)_3_ suspension in 2-propanol was separated from the aqueous phase and diluted with water in order to achieve a water-2-propanol ratio 0.25. PMMA was added to the suspension and dissolved at 50 °C.

Pure PMMA polymer solutions and FRM suspensions, containing dissolved PMMA, were used for dip coating of 304 type stainless steel foils. In a typical dip coating procedure, a substrate was immersed in the PMMA solution without FRM or with dispersed FRM, remained in the solution for 30 s, and then it was withdrawn from the solution at a constant speed of 1 mm s^−1^. Testing was performed using a microscope JEOL SEM, Japan, (JSM-7000F), Bruker USA (D8, Cu-Kα) X-ray diffractometer and FTIR, Vertex USA (Vertex 70) spectrometer. Coating adhesion was tested according to ASTM D3359-17 for comparison with adhesion of other polymer coatings [42,43,44]. The experimental details and equipment for corrosion testing in 3% NaCl solutions were described in [45,46].

## 4. Conclusions

This study demonstrated that despite the PMMA insolubility in 2-propanol and water, high M_W_ PMMA was well-soluble in their mixtures with different water contents. The use of high M_W_ PMMA and high solution concentration were key factors for the PMMA deposition by a dip coating method. The use of carcinogenic and toxic solvents for PMMA can be eliminated. The amount of the deposited PMMA can be changed by the variation of the PMMA concentration, solvent composition, and number of the deposited layers. PMMA coatings were used for the protection of stainless steel from aqueous corrosion. PMMA deposits were loaded with environmentally friendly FRM, such as commercial halloysite, huntite, and hydrotalcite, as well as synthesized Al(OH)_3_, avoiding the use of toxic halogen-based FRM. A fundamentally new strategy was developed for the fabrication of PMMA-Al(OH)_3_ coatings, which was based on the salting out aided dispersive extraction of Al(OH)_3_ particles. HDPA is a promising extractor for the particle transfer. The method allows for reduced particle agglomeration. The ability to deposit multiple PMMA layers and composite layers indicates that the dip coating method can be applied for the fabrication of multilayer coatings, containing different layers and for the deposition of coatings of graded composition. The method can potentially be applied for the deposition of composites, containing other functional organic and inorganic materials.

## Data Availability

The data presented in this study are available in: Poly(methyl methacrylate) coatings containing flame retardant additives from suspensions in water-2-propanol.
